# Prognostic impact of urokinase-type plasminogen activator receptor (uPAR) in cytosols and pellet extracts derived from primary breast tumours

**DOI:** 10.1054/bjoc.2001.1867

**Published:** 2001-07

**Authors:** J H de Witte, J A Foekens, N Brünner, J J T M Heuvel, ThH van Tienoven, M P Look, J G M Klijn, A Geurts-Moespot, N Grebenchtchikov, ThJ Benraad, C G J Sweep

**Affiliations:** Department of Chemical Endocrinology, University Medical Centre Nijmegen, Nijmegen, The Netherlands; Division of Endocrine Oncology (Department of Medical Oncology), Rotterdam Cancer Institute (Daniel den Hoed Kliniek) and University Hospital Rotterdam, Rotterdam, The Netherlands; The Finsen Laboratory, Rigshospitalet, Copenhagen, Denmark

## Abstract

Using a previously developed enzyme-linked immunosorbent assay (ELISA), the levels of the receptor for urokinase-type plasminogen activator (uPAR) were determined in cytosols and corresponding membrane pellets derived from 878 primary breast tumours. The levels of uPAR in the pellet extracts were more than 3-fold higher than those measured in the cytosols (*P*< 0.001). Moreover, the uPAR levels in the two types of extracts were weakly, though significantly, correlated with each other (r_S_= 0.20, *P*< 0.001). In Cox univariate analysis, high cytosolic levels of uPAR were significantly associated with reduced overall survival (OS) and relapse-free survival (RFS). The levels of uPAR in pellet extracts appeared not to be related with patient survival. In multivariate analysis, elevated levels of uPAR measured in cytosols and pellet extracts were found to be independent predictors of poor OS, not RFS. The prediction of poor prognosis on the basis of high uPAR levels emphasizes its important role in plasmin-mediated degradation of extracellular matrix proteins during cancer invasion and metastasis. © 2001 Cancer Research Campaign http://www.bjcancer.com


British Journal of Cancer (2001) **85**(1), 85–92
© 2001 Cancer Research Campaign
doi: 10.1054/ bjoc.2001.1867, available online at http://www.idealibrary.com on IDEAL®

http://www.bjcancer.com

### Prognostic impact of urokinase-type plasminogen activator receptor (uPAR) in cytosols and pellet extracts derived from primary breast tumours

**JH de Witte[1], JA Foekens[2], N Brünner[3], JJTM Heuvel[1], ThH van Tienoven[1], MP Look[2], JGM Klijn[2], A Geurts-Moespot[1], N Grebenchtchikov[1], ThJ Benraad[1] and CGJ Sweep[1]**

[1]Department of Chemical Endocrinology, University Medical Centre Nijmegen, Nijmegen, The Netherlands; [2]Division of Endocrine Oncology (Department of Medical Oncology), Rotterdam Cancer Institute (Daniel den Hoed Kliniek) and University Hospital Rotterdam, Rotterdam, The Netherlands; [3]The Finsen Laboratory, Rigshospitalet, Copenhagen, Denmark

**Summary** Using a previously developed enzyme-linked immunosorbent assay (ELISA), the levels of the receptor for urokinase-type plasminogen activator (uPAR) were determined in cytosols and corresponding membrane pellets derived from 878 primary breast tumours. The levels of uPAR in the pellet extracts were more than 3-fold higher than those measured in the cytosols ($P < 0.001$). Moreover, the uPAR levels in the two types of extracts were weakly, though significantly, correlated with each other ($r_s = 0.20$, $P < 0.001$). In Cox univariate analysis, high cytosolic levels of uPAR were significantly associated with reduced overall survival (OS) and relapse-free survival (RFS). The levels of uPAR in pellet extracts appeared not to be related with patient survival. In multivariate analysis, elevated levels of uPAR measured in cytosols and pellet extracts were found to be independent predictors of poor OS, not RFS. The prediction of poor prognosis on the basis of high uPAR levels emphasizes its important role in plasmin-mediated degradation of extracellular matrix proteins during cancer invasion and metastasis. © 2001 Cancer Research Campaign http://www.bjcancer.com

During cancer invasion and metastasis, tumour cells actively traverse host cellular and extracellular matrix barriers (Mignatti and Rifkin, 1993). Proteolytic enzymes of the plasminogen activation system released by the neoplastic cells and/or the surrounding tumour stroma accomplish invasion into the surrounding normal tissue by degradation of basement membranes and extracellular matrix proteins (Liotta et al, 1982; Danø et al, 1985; Mignatti and Rifkin, 1993). Central to this system is the conversion of the abundant zymogen plasminogen into active plasmin, which is able to degrade several extracellular proteins and to activate latent prometalloproteases (Duffy, 1992; Mignatti and Rifkin, 1993; Andreasen et al, 1997). The activation of plasminogen can be catalysed by urokinase-type plasminogen activator (uPA) and tissue-type plasminogen activator (tPA), the activities of both enzymes being controlled by 2 plasminogen activator inhibitors, PAI-1 and PAI-2 (Andreasen et al, 1990, 1997). Binding of uPA to its specific cellular receptor, uPAR, is considered to be an important step in the proteolytic cascade, since this receptor localizes the proteolytic activities to the cell surface and strongly enhances activation of surface-bound plasminogen (Danø et al, 1994).

Human uPAR is a heavily glycosylated protein of approximately 60 kDa comprising three homologous domains (Behrendt et al, 1995). The receptor is anchored in the plasma membrane by a carboxyl-terminally attached glycosyl-phosphatidylinositol moiety which is responsible for the hydrophobicity of the protein (Ploug et al, 1991). Primarily, uPA binds to the amino-terminal domain of uPAR, while other domains appear to critically contribute to strong ligand binding (Behrendt et al, 1996). It has been shown that uPAR acts as a high-affinity receptor for the matrix-like form of vitronectin also (Waltz and Chapman, 1994; Wei et al, 1994; Kanse et al, 1996). This function of uPAR, together with its ability to interact with the cytoskeleton-engaged integrins (Wei et al, 1996), implicate uPAR in regulation of cell adhesion and migration, in addition to mediation of cellular signalling (Chapman, 1997).

*Received 12 December 2000*
*Revised 19 March 2001*
*Accepted 27 March 2001*

*Correspondence to:* CGJ Sweep

Many studies have shown that uPA and PAI-1 are prognostic factors in several types of cancer, including breast cancer, high tissue levels of both components being associated with reduced disease-free and/or overall survival (reviewed by Duffy, 1996 and Schmitt et al, 1997). Relatively few studies have reported on the clinical relevance of uPAR in human cancer, though elevated levels of uPAR have been shown to predict poor disease outcome in breast cancer as well (Duggan et al, 1995; Grøndahl-Hansen et al, 1995; Bouchet et al, 1999; Foekens et al, 2000). Recently, we evaluated the clinical relevance of uPA, tPA and PAI-1 determined by a newly developed ELISA in cytosolic extracts as well as in corresponding detergent extracts of pellets obtained after ultracentrifugation when preparing the cytosolic fractions (De Witte et al, 1999a, b). In the present study, we measured the antigen levels of uPAR in the same cytosols and pellet extracts, employing a previously established ELISA (Rønne et al, 1995). Using both subcellular fractions enables us to analyse direct correlations between all components of the urokinase system and to weigh them against each other. The quantitative ELISA results obtained were compared and the prognostic information provided by uPAR in these specimens was examined.

### MATERIALS AND METHODS

### Patients and tumour characteristics

In the present study, only those 878 patients with primary operable breast cancer were included for whom uPAR antigen levels were measured in both cytosols and pellet extracts derived from breast tumour biopsies. Inclusion criteria for patients from whom tumour samples were stored in the tumour bank of the Rotterdam Cancer Institute (Dr Daniel den Hoed Kliniek) were: (i) primary diagnosis of breast cancer between 1979 and 1989; (ii) no signs of distant metastasis at diagnosis; (iii) no previous diagnosis of carcinoma, with the exception of basal cell skin cancer and cervical cancer stage Ia; (iv) no evidence of disease within one month after primary surgery. In case of mastectomy after an initial lumpectomy for residual disease, the mastectomy is considered (as part of) primary treatment. The median number of lymph nodes removed surgically was 11. Patients without primary surgery or patients who received neo-adjuvant treatment before primary surgery, were excluded. Median age of the patients at the time of surgery was 56 years (range 25–89 years). Radiotherapy was given to 82% of the patients: on the breast/thoracic wall to 608 patients and/or on the axilla to 259 patients, and/or on one or more lymph node areas other than the axilla to 295 patients. While 2 of the 439 node-negative patients received adjuvant chemotherapy, none of them received adjuvant hormonal therapy. Of the 430 node-positive patients (for 9 patients, nodal status missing), adjuvant chemotherapy (mainly CMF; cyclophosphamide, methotrexate, 5-fluorouracil) was given to 154 patients, while 58 patients received hormonal therapy, either alone (45 patients) or in combination with chemotherapy (13 patients). All patients were routinely examined every 3 to 6 months during the first 5 years of follow-up and once a year thereafter.

During follow-up, 363 patients (41%) showed relapse, and counted as failures in the analysis for relapse-free survival. 51 patients (7%) died without evidence of disease and were censored at last follow-up in the analysis for relapse-free survival. 221 patients (25%) died after a previous relapse. A total of 282 patients (32%) were counted as failures in the analysis for overall survival. The median follow-up period of patients alive was 100 months (range 12–167 months).

### Tumour tissue extraction

Tumour tissues were stored in liquid nitrogen and pulverized in the frozen state with a microdismembrator as recommended by the European Organization for Research and Treatment of Cancer (EORTC Breast Cancer Co-operative Group, 1980) for processing of breast tumour tissue for cytosolic determination of steroid hormone receptors (ER and PgR). The resulting tissue powder was homogenized (weight/volume: 1/6) in EORTC receptor buffer (10 mM $K_2HPO_4$, containing 1.5 mM di-potassium chloride EDTA, 3 mM sodium azide, 10 mM monothioglycerol and 10% (v/v) glycerol, pH = 7.4). The homogenate was centrifuged for 30 min at 100 000 ***g*** and 4˚C to obtain the supernatant fraction (cytosol). The 100 000 ***g*** pellets were re-homogenized with an Ultraturrax tissue homogenizer in 20 mM Tris/HCl (pH = 8.5) containing 125 mM NaCl. Following the addition of Triton X-100 to a final concentration of 1% and subsequent incubation for 16–20 h at 4˚C, the supernatant fractions obtained by centrifugation at 30 000 ***g*** at 4˚C were designated as pellet extracts.

### Steroid hormone receptor assays

ER and PgR levels were determined by ligand-binding assay or enzyme immunoassay in cytosols as described previously (Foekens et al, 1989). The cut-off level used as to classify tumours as ER and PgR positive or negative was 10 fmol $mg^{-1}$ cytosolic protein.

### uPAR ELISA

The antigen levels of uPAR in cytosols and pellet extracts were assessed with an ELISA as described by Rønne and colleagues (1995), with some minor modifications. Briefly, microtitreplates were coated overnight at 4˚C with rabbit anti-uPAR antibodies. Bound ligand was detected by subsequent incubation with non-biotinylated mouse monoclonal anti-uPAR antibodies (clone R2, R3 and R5) followed by incubation with horse-radish peroxidase-labelled goat anti-mouse antibodies (Sigma Chemical Co, St Louis, MO) diluted 1:500. Recombinant soluble uPAR was used as standard in the ELISA (Rønne et al, 1994). The uPAR ELISA detects free uPAR and uPAR in complex with uPA (Rønne et al, 1994).

ELISA reagents were all diluted in PBS with 1% BSA and 0.1% (v/v) Tween-20. Incubations with standards, samples and controls, diluted in PBS-BSA-Tween, were performed overnight at 4˚C. All determinations were performed in duplicate. Triton X-100 did not have any influence on the ELISA up to a concentration of 1%. The reproducibility of assay performance was controlled by analysis of an aliquot of a pooled breast tumour cytosol sample in each assay-run and the between-assay variation was found to be below 10%. The within-assay variation of samples measured in duplicate was always below 5%. The detection limit of the assay was 24 pg $ml^{-1}$.

### Protein determinations

The Bradford method for protein analysis (Bradford, 1976) was employed using the Bio-Rad reagent with human serum albumin (KabiVitrum, Stockholm, Sweden) as a standard in order to express antigen levels per mg of total protein. Triton X-100 up to a concentration of 1% did not interfere with the protein determination in pellet extracts.

### Statistical analysis

The strength of the associations of uPAR antigen levels determined in cytosols and pellet extracts with age and steroid hormone receptor status was tested with Spearman rank correlation ($r_s$). The associations of uPAR with other clinical variables were tested with the non-parametric Wilcoxon Rank-Sum test or the Kruskal-Wallis test, including a Wilcoxon-type test for trends across ordered groups where appropriate. Relapse-free and overall survival probabilities were calculated by the actuarial method of Kaplan and Meier (Kaplan and Meier, 1958). Both uni- and multivariate analyses were performed using the Cox proportional hazard model, and the associated likelihood ratio test was used to test for differences. The Log-rank test for trends was used to test ordered variables. To evaluate the prognostic value of uPAR in addition to classical prognostic factors, a basic model was introduced. The prognostic significance of additional biochemical variables (uPA, tPA, and PAI-1) was tested in a final model, in which the basic model and all of the biochemical variables were included. All computations were done with the STATA statistical package, release 6.0 (STATA Corp, College Station, TX). Two-sided $P$-values below 0.05 were considered to be statistically significant.

© 2001 Cancer Research Campaign

### RESULTS

### uPAR in cytosols and pellet extracts

The antigen levels of uPAR were determined in cytosols and corresponding pellet extracts derived from primary breast cancer patients. The cytosolic levels of uPAR varied from 0.00 to 9.88 ng $mg^{-1}$ protein (median 0.99 ng $mg^{-1}$ protein; mean ± SD 1.13 ± 0.85 ng mg $protein^{-1}$). The levels of uPAR in the pellet extracts were significantly higher ($P < 0.001$) than those measured in the cytosols and ranged from 0.00 to 204.90 ng $mg^{-1}$ protein (median 3.38 ng $mg^{-1}$ protein; mean ± SD 4.95 ± 10.58 ng mg $protein^{-1}$). The levels of uPAR in cytosols and pellet extracts were weakly but significantly correlated ($r_s = 0.20$, $P < 0.001$) (Figure 1).

### Relation of uPAR to patient and tumour characteristics

The levels of uPAR determined in cytosols and pellet extracts were related to patient and tumour characteristics (Table 1). The uPAR levels were not or only marginally, negatively related with patient's age. The uPAR levels in cytosols or pellet extracts were not related with menopausal status and grade of differentiation. In contrast to the levels of uPAR in cytosols, those measured in the pellet extracts were negatively associated with both tumour size and nodal status. Overall, higher levels of uPAR were measured in steroid hormone receptor-negative tumours. The Spearman correlation coefficients found for the cytosols were weak, i.e. $r_s = -0.22$ for uPAR and ER, and $r_s = -0.12$ for uPAR and PgR. For the pellet extracts these correlation coefficients were weak as well, i.e. –0.13 and –0.07, respectively. In both the cytosols and the pellet extracts uPAR levels were strongly positively correlated with those of uPA and PAI-1 in the respective sample, with Spearman correlations ranging from +0.42 to +0.63. In the cytosols, uPAR levels were weakly negatively correlated with those of tPA ($r_s = -0.12$) while in the pellet extracts the relationship between uPAR and tPA was weakly positive ($r_s = +0.10$).

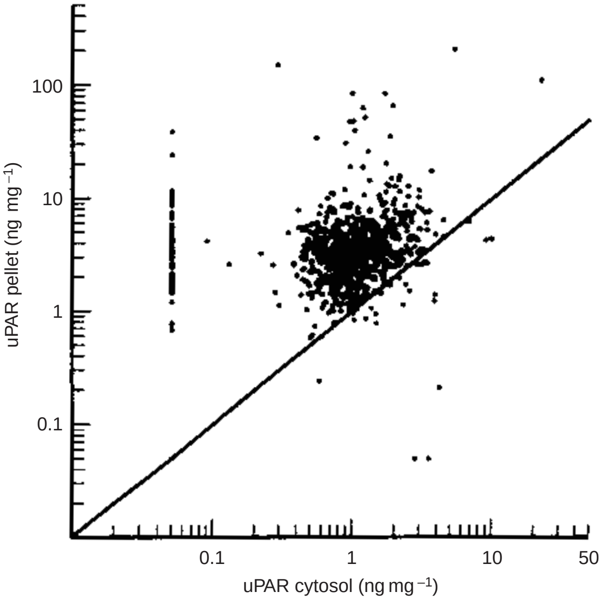

**Figure 1** Correlation of uPAR antigen levels determined in cytosols and pellet extracts derived from primary breast cancer patients. The solid line corresponds to the line of equality. Values below the assay sensitivity were set at half the detection limit (0.05 ng $ml^{-1}$) of the ELISA. The antigen levels are expressed as ng $mg^{-1}$ of total protein content measured in the cytosols and pellet extracts

### Relation of uPAR to survival

In order to analyse the relationship between uPAR and patient's survival in univariate analysis, the antigen levels were divided into 4 groups ($Q_1$ to $Q_4$) by their respective quartiles. Regarding the cytosolic extracts, the best value for discriminating between patients with short and long survival corresponded to the third quartile of the uPAR antigen levels (Table 2). Since there were no significant associations between the uPAR levels in pellet extracts and the rate of relapse and death (Table 2), the median value of the respective uPAR levels was arbitrarily chosen to divide patients into a 'low-risk' and a 'high-risk' group. When using these values as cut-off points in repeated univariate analyses, high levels of uPAR in cytosols (i.e. in $Q_4$) were found to be associated with a significantly reduced RFS and OS (Figure 2A, C). In contrast, high levels of uPAR determined in pellet extracts (i.e. above the median) appeared not to be significantly related to RFS or OS (Figure 2B, D).

The combined prognostic value of the cytosolic levels of uPAR and of uPA, the latter previously determined in the same samples (De Witte et al, 1999a), with respect to RFS and OS is visualized in Figure 3. Patients with high levels of uPAR (i.e. in $Q_4$) in their tumours, which, in addition, contain high levels of uPA (i.e. above the median value) experienced a very poor prognosis, while patients with both low uPAR and low uPA tumour levels encountered a favourable prognosis. No further discrimination between high-risk and low-risk patients was obtained when combining the categorized levels of uPAR and uPA in pellet extracts (results not shown).

Cox multivariate regression analysis was performed to compare the prognostic significance of uPAR with that of the classical prognostic parameters comprising the basic multivariate model. As reported earlier (De Witte et al, 1999), in the basic model, age menopausal status, lymph node status, ER status and adjuvant therapy were all significantly associated with both RFS and OS. However, tumour size and PgR status were only statistically significant in predicting OS, not RFS (results not shown). When added to the basic multivariate model, uPAR measured in cytosols ($Q_4$ versus $Q_1$–$Q_3$) contributed to the prognostic information in the analysis for OS, but not in the analysis for RFS. Likewise, uPAR in pellet extracts (above versus below the median value) added to the basic multivariate model for OS, but not for RFS (Table 3).

A final model was analysed which included the basic multivariate model, the antigen levels of uPAR ($Q_4$ versus $Q_1$–$Q_3$), as well as the levels of uPA, tPA and PAI-1 (above versus below the median values) determined previously in either the cytosols or the pellet extracts (De Witte et al, 1999a,b). In the multivariate analysis with the cytosolic variables, cytosolic PAI-1 appeared as the only independent variable in analysis for RFS, while both uPA and PAI-1 were associated with reduced OS. In the multivariate model with the biochemical variables determined in the pellet extracts, PAI-1 as well as tPA were associated with a reduced (PAI-1) or prolonged (tPA) RFS and OS (Table 3).

### DISCUSSION

In the present study we analysed uPAR antigen levels in 878 breast tumour cytosols and corresponding detergent extracts of high-speed

**Table 1** Relationships of uPAR antigen levels in 878 cytosols and pellet extracts with patient and tumour characteristics

| Characteristics | Number of patients[1] | Percentage of tumours above the median value[2] | |
|---|---|---|---|
| | | $uPAR_{cytosol}$[3] | $uPAR_{pellet}$[3] |
| All patients | 878 | 50 | 50 |
| Age at surgery (y) | | | |
| ≤40 | 122 | 52 | 55 |
| 41–55 | 301 | 51 | 50 |
| 56–70 | 290 | 50 | 51 |
| >70 | 165 | 44 | 45 |
| *P* value[4] | | 0.04 | 0.09 |
| Menopausal status | | | |
| Pre-menopausal | 361 | 51 | 51 |
| Post-menopausal | 517 | 48 | 50 |
| *P* value[5] | | 0.07 | 0.41 |
| Tumour size | | | |
| $T_1$ (≤2 cm) | 397 | 48 | 56 |
| $T_2$ (>2–5 cm) | 396 | 52 | 46 |
| $T_{3/4}$ (>5 cm) | 85 | 46 | 42 |
| *P* value[6] | | 0.51 | <0.001 |
| Nodal status | | | |
| $N_0$ | 439 | 51 | 54 |
| $N_{1–3}$ | 211 | 46 | 49 |
| $N_{>3}$ | 219 | 48 | 42 |
| *P* value[6] | | 0.99 | 0.003 |
| Grade | | | |
| Well/moderate | 151 | 48 | 53 |
| Poor | 503 | 49 | 51 |
| *P* value[5] | | 0.84 | 0.68 |
| ER positive[7] | | | |
| No | 207 | 64 | 60 |
| Yes | 670 | 45 | 47 |
| *P* value[4] | | <0.001 | <0.001 |
| PgR positive[7] | | | |
| No | 248 | 57 | 57 |
| Yes | 616 | 46 | 48 |
| *P* value[4] | | 0.001 | 0.03 |
| uPA[8] | | | |
| Low | 434/434[9] | 30 | 27 |
| High | 444/435[9] | 69 | 72 |
| *P* value[4] | | <0.001 | <0.001 |
| PAI-1[8] | | | |
| Low | 435/439[9] | 34 | 32 |
| High | 443/434[9] | 64 | 68 |
| *P* value[4] | | <0.001 | <0.001 |
| tPA[8] | | | |
| Low | 434/435[9] | 53 | 46 |
| High | 437/430[9] | 46 | 54 |
| *P* value[4] | | <0.001 | 0.002 |

[1]Due to missing values the numbers do not always add up to 878. [2]The median values in ng mg protein$^{-1}$ were 0.99 for $uPAR_{cytosol}$ and 3.38 for $uPAR_{pellet}$. [3]$uPAR_{cytosol}$ and $uPAR_{pellet}$ denotes uPAR levels determined in cytosol and pellet extracts, respectively. [4]*P* value for Spearman rank correlation. [5]*P* value for Wilcoxon rank sum test (for grade: well and moderate combined). [6]*P* value for Kruskal-Wallis test, including a Wilcoxon-type test for trend. [7]Cut-off point used for ER and PgR: 10 fmol mg protein$^{-1}$. [8]Median values used to classify tumours as high and low in the cytosols and pellet extracts were as published before; for uPA, 0.73 and 7.26 ng mg protein$^{-1}$ (de Witte et al, 1999a), for PAI-1, 1.62 and 5.29 ng mg protein$^{-1}$ (de Witte et al, 1999a), and for tPA, 2.40 and 13.01 ng mg protein$^{-1}$ (de Witte et al, 1999b). These biochemical assays are very sensitive and easily can be performed on as little as 25 mg of tissue. [9]The number of cytosols/pellet extracts.

pellets employing a slightly modified version of a previously developed ELISA. This ELISA has also been used before to evaluate the clinical relevance of uPAR in primary breast tumour (Grøndahl-Hansen et al, 1995; Foekens et al, 2000). In both studies, uPAR antigen levels were measured in cytosols routinely prepared for steroid hormone receptor determination, while Grøndahl-Hansen et al (1995) also measured uPAR in corresponding detergent-treated homogenates of the same breast tumour samples.

The cytosolic levels of uPAR found in the present study were highly comparable to the cytosolic levels reported by Grøndahl-Hansen et al (1995) and Foekens et al (2000) (median: 0.87 versus 0.99 and 0.94 ng mg$^{-1}$, respectively). Evidently, this finding is a

 *© 2001 Cancer Research Campaign*

direct reflection of the application of the same ELISA to the same type of tumour extracts. The presence of uPAR in the cytosolic samples prepared with a detergent-free, neutral extraction buffer, most probably represents a water-soluble form of native uPAR which has lost its glycolipid membrane anchor. As has been suggested earlier, soluble isoforms of membrane proteins like uPAR may be generated by alternative RNA splicing or by cleavage of a region between the membrane anchor and the mature integral membrane protein by limited proteolysis (Ploug et al, 1992). Alternatively spliced mRNA variants have been identified and characterized in both human and mouse cell lines and tissues (Kristensen et al, 1991; Pyke et al, 1993). However, although the

**Table 2** Cox univariate analyses of relapse-free and overall survival in 878 primary breast cancer patients as a function of uPAR levels in cytosols and pellet extracts

| Factor | Relapse-free survival | | Overall survival | |
|---|---|---|---|---|
| | *P* value | RHR[1] (95% CI) | *P* value | RHR[1] (95% CI) |
| uPAR *cytosols* | | | | |
| $Q_2$ vs. $Q_1$ | 0.68 | 1.11 (0.82–1.50) | 0.85 | 1.03 (0.73–1.46) |
| $Q_3$ vs. $Q_1$ | 0.69 | 1.06 (0.79–1.44) | 0.54 | 1.11 (0.79–1.57) |
| $Q_4$ vs. $Q_1$ | 0.050 | 1.34 (1.00–1.79) | 0.033 | 1.43 (1.03–1.98) |
| uPAR *pellet extracts* | | | | |
| $Q_2$ vs. $Q_1$ | 0.62 | 0.93 (0.70–1.24) | 0.26 | 0.82 (0.58–1.16) |
| $Q_3$ vs. $Q_1$ | 0.73 | 0.95 (0.71–1.27) | 0.45 | 1.13 (0.82–1.56) |
| $Q_4$ vs. $Q_1$ | 0.75 | 1.05 (0.79–1.40) | 0.45 | 1.13 (0.82–1.57) |

[1]Relative hazard rate (RHR) with 95% confidence interval (CI). uPAR antigen levels in ng $mg^{-1}$ protein, classified as quarters ($Q_1$–$Q_4$): quartiles 0.69, 0.99 and 1.41 for cytosols and 2.26, 3.38 and 4.82 for pellet extracts.

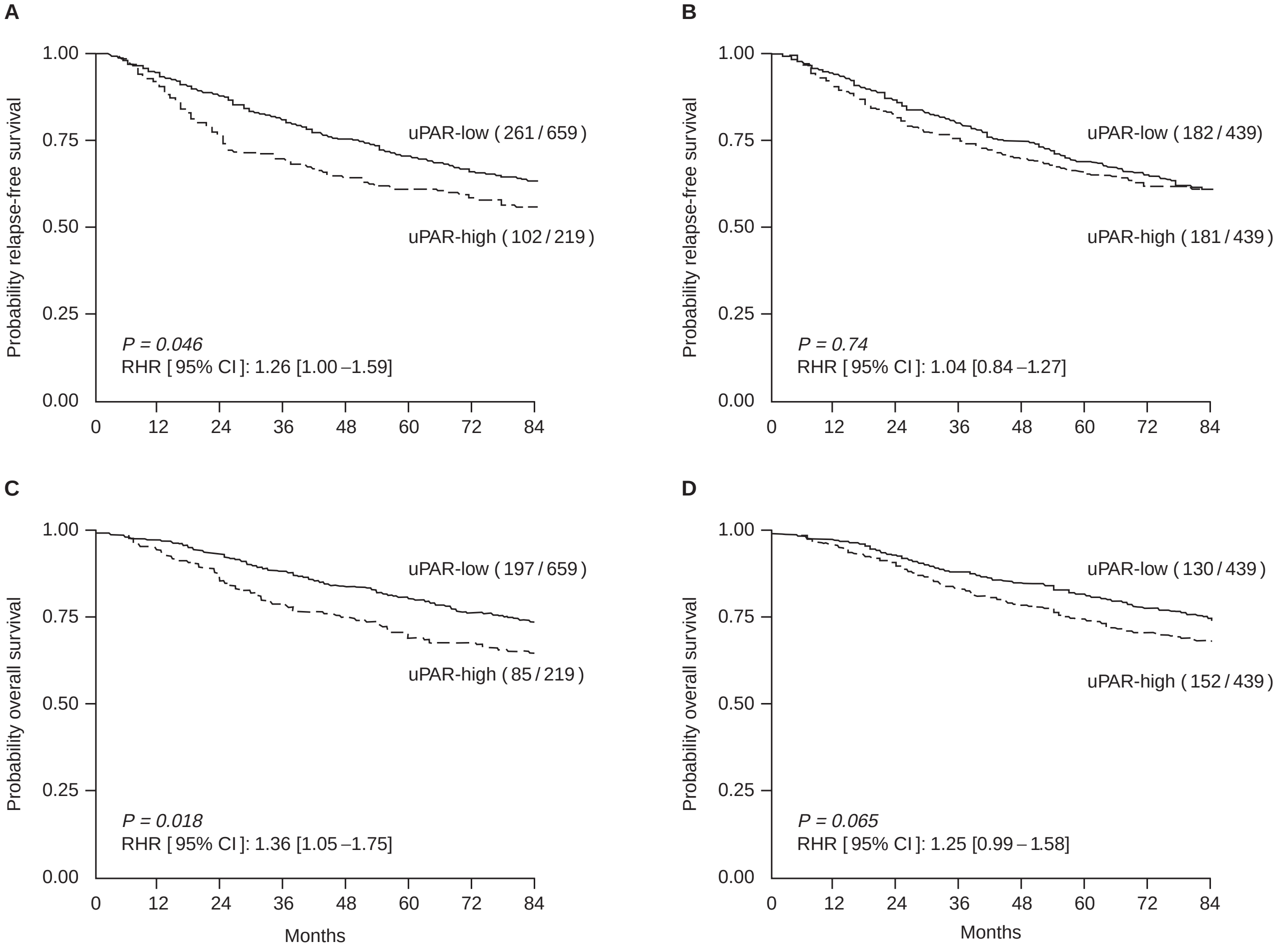

**Figure 2** Actuarial relapse-free and overall survival curves for all 878 breast cancer patients stratified by the levels of uPAR determined in cytosols (**A** and **C**) and pellet extracts (**B** and **D**). Patients were divided into groups with uPAR levels below ('uPAR low') and above ('uPAR high') the third quartile (cytosols; cut-off point 1.41 ng $mg^{-1}$) or median value (pellet extracts; cut-off point 3.38 ng $mg^{-1}$) as indicated in the legend to Table 2. Shown are the *P* values from the Log-rank test for trend. RHR (95% CI), relative hazard rate (95% confidence interval) calculated by Cox regression analysis. Between parentheses the number of failures/total number of patients in each group

*© 2001 Cancer Research Campaign*

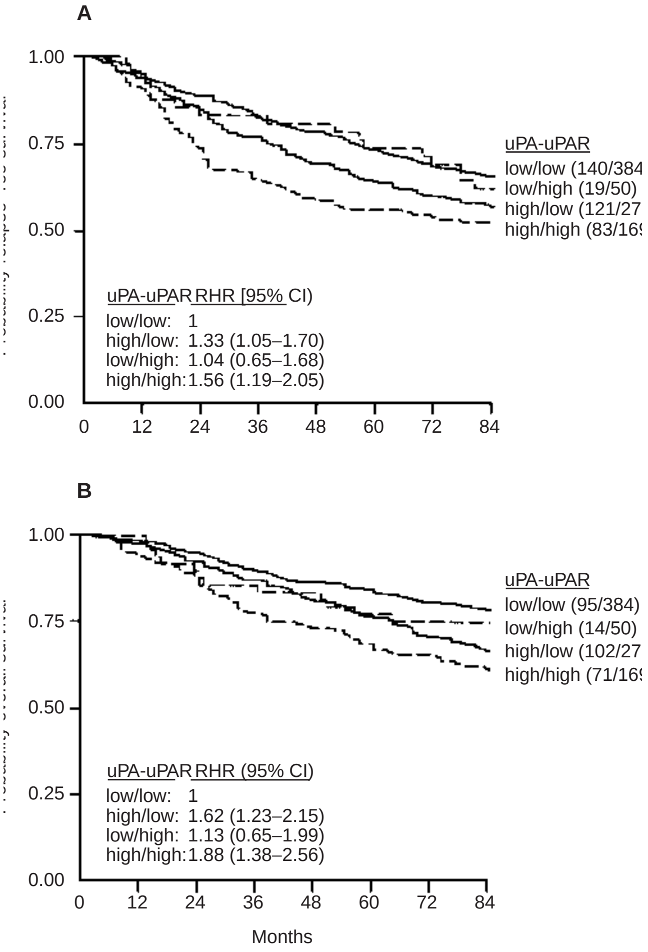

**Figure 3** Actuarial relapse-free (**A**) and overall survival curves (**B**) for patients stratified by the combined levels of uPAR and uPA determined in cytosolic extracts of tumour tissue. Patients were divided into groups with levels below ('low') or above ('high') the third quartile (uPAR levels in $Q_4$) or median value (uPA) of the respective antigen levels. RHR (95% CI), relative hazard rate (95% confidence interval) calculated by Cox univariate regression analysis. Between parentheses the number of failures/total number of patients in each group deduced protein molecules contain a uPA binding domain and lack the glycolipid anchor attachment sites, expression of the corresponding native proteins has not been demonstrated under natural conditions. Therefore, a more plausible explanation for its presence in cytosols may be related to the pericellular proteolytic activities mediated by uPAR during cancer invasion and metastasis. Under these conditions, uPAR is likely to be exposed to a proteolytic micro-environment in vivo where it may be solubilized by phospholipases or proteases, like plasmin, and even may enter the bloodstream, as has been implied by several authors (Ploug et al, 1992; Grøndahl-Hansen et al, 1995; Pappot et al, 1997; Stephens et al, 1997). Indeed, recent publications have shown that by measuring the soluble form of uPAR in peripheral blood important prognostic information in patients with colon (Stephens et al, 1999) or ovarian cancer (Sier et al, 1998) can be obtained. We are currently studying the prognostic role of soluble uPAR in blood samples obtained from patient undergoing surgery for primary breast cancer.

The detergent-treated homogenates of breast tumour tissue used by Grøndahl-Hansen and colleagues (1995) need to be distinguished from the pellet extracts in this investigation. While the detergent-treated homogenates most likely contain the lipophilic native as well as the soluble form of uPAR, the pellet extracts should contain exclusively the former variant of uPAR. Nevertheless, the levels of uPAR determined in the pellet extracts were substantially higher than those in the detergent-treated homogenates (median: 3.38 versus 1.66 ng $mg^{-1}$ protein, mean 4.95 versus 1.91 ng $mg^{-1}$ protein). Since different procedures were employed to prepare the detergent-treated homogenates and the pellet extracts, a direct comparison of the uPAR antigen levels measured is quite difficult. However, the apparent discrepancy may be explained, in part, by the higher total protein content in the detergent extracts (3.14 mg $ml^{-1}$) derived from the tumour homogenates as compared to the pellet extracts (1.92 mg $ml^{-1}$) in the present study, causing relatively lower uPAR antigen levels in the former type of samples.

In this study, the prognostic value of uPAR in primary breast carcinomas as documented in earlier investigations by Duggan et al (1995). Grøndahl-Hansen et al (1995), Bouchet et al (1999) and Foekens et al (2000) could be confirmed, high levels of uPAR being associated with poor disease outcome. In univariate regression analysis, using the third quartile of the cytosolic uPAR levels as a cut-off point, cytosolic uPAR was shown to predict a significantly reduced RFS and especially OS in patients with elevated levels of uPAR (Figure 2). In contrast, the uPAR levels in the pellet extracts were not significantly related to patient's survival (Figure 2). In multivariate analysis, after correction for the classical prognostic factors, high levels of uPAR determined in both cytosols (i.e. in $Q_4$) and pellet extracts (i.e. above the median value) appeared as significant predictors of poor OS, but not RFS (Table 3). These findings are in agreement with the results obtained in the study by Grøndahl-Hansen et al (1995) referred to above. In this latter investigation, uPAR in cytosols and detergent-treated homogenates appeared to be predictive of a shorter OS only, the cytosolic samples providing the most pronounced prognostic information.

Amongst others, we have previously demonstrated that uPA, PAI-1 and tPA determined either in cytosols or pellet extracts derived from the same series of patients included in the present study can be considered as independent prognostic markers (i.e. independent of classical prognostic factors) in breast cancer (De Witte et al, 1999a,b). When these biochemical variables in addition to uPAR, were included together with the classical prognostic variables in multivariate models, PAI-1 in cytosols and pellet extracts appeared to be the strongest biological prognostic parameter for RFS and OS (Table 3). This finding is in general agreement with various other studies on the clinical relevance of components of the plasminogen activation system. Although different combinations of potential prognostic variables have been included in the multivariate analyses performed in those studies, the strong prognostic impact of PAI-1 antigen in breast cancer has been consistently reported (see review by Schmitt et al, 1997; Look and Foekens, 1999).

The association of elevated tumour levels of uPAR with poor prognosis is in good agreement with the proposed role of this molecule in cancer invasion and metastasis. Indeed, uPA-mediated degradation of extracellular matrix proteins is strongly enhanced upon binding of uPA to uPAR (Ellis et al, 1991). In the present study, the significance of the interaction of uPA with uPAR is expressed by the observed powerful negative effect on disease outcome when the cytosolic levels of both parameters were

 *© 2001 Cancer Research Campaign*

**Table 3** Cox multivariate analyses of relapse-free and overall survival in primary breast cancer patients

| Factor | Relapse-free survival | | Overall survival | |
|---|---|---|---|---|
| | *P* value | RHR[1] (95% CI) | *P* value | RHR[1] (95% CI) |
| Basic multivariate model | | | | |
| + uPAR *cytosols*[2] | 0.10 | 1.22 (0.96–1.56) | 0.03 | 1.35 (1.03–1.78) |
| + uPAR *pellet extracts*[3] | 0.24 | 1.14 (0.92–1.41) | 0.005 | 1.42 (1.11–1.82) |
| Final multivariate model | | | | |
| *Cytosols*[2] | | | | |
| uPA | 0.22 | 1.17 (0.91–1.50) | 0.01 | 1.44 (1.09–1.91) |
| tPA | 0.69 | 0.95 (0.76–1.20) | 0.83 | 0.97 (0.74–1.27) |
| PAI-1 | 0.006 | 1.42 (1.11–1.81) | 0.006 | 1.49 (1.12–1.97) |
| uPAR | 0.71 | 1.05 (0.81–1.35) | 0.65 | 1.07 (0.80–1.43) |
| *Pellet extracts*[3] | | | | |
| uPA | 0.99 | 1.00 (0.78–1.28) | 0.33 | 1.15 (0.86–1.54) |
| tPA | 0.03 | 0.77 (0.61–0.98) | 0.002 | 0.64 (0.49–0.84) |
| PAI-1 | 0.003 | 1.47 (1.14–1.88) | 0.007 | 1.48 (1.12–1.97) |
| uPAR | 0.94 | 1.01 (0.79–1.29) | 0.07 | 1.29 (0.98–1.71) |

[1]Relative hazard rate (RHR) with 95% confidence interval (CI) of multivariate analyses corrected for the basic model including age/menopausal status, tumour size, nodal status, ER/PgR status and adjuvant therapy (upper part of the table). The final multivariate model included the basic model in addition to the antigen levels of uPA, tPA, PAI-1 and uPAR determined either in cytosols or pellet extracts. [2]Antigen levels of the respective components determined in cytosols in ng mg protein$^{-1}$ divided by their median levels (uPA, tPA, PAI-1) or third quartile (uPAR). [3]Antigen levels of the respective components determined in pellet extracts in ng mg protein$^{-1}$ divided by their median levels (uPA, tPA, PAI-1 and uPAR).

combined (Figure 3). The uPAR ELISA employed in the present study measures both the free, uncomplexed (soluble) form of uPAR as well as (soluble) uPAR in complex with uPA, without having the ability to distinguish between them (Rønne et al, 1995). Considering the importance of the interplay between uPA and uPAR with respect to the invasiveness of malignant cells, the tumour levels of complexes between uPA and uPAR might represent an even stronger prognostic parameter than the levels of the separate components. Therefore, the development of an ELISA for the selective detection and quantitation of uPA:uPAR complexes in tumour tissue extracts (De Witte et al, 1997) might be considered highly relevant to the analysis of the potential prognostic impact of uPA:uPAR complexes in breast cancer.

### REFERENCES

Andreasen PA, Georg B, Lund LR, Riccio A and Stacey SN (1990) Plasminogen activator inhibitors: hormonally regulated serpins. *Mol Cell Endocrinol* **68**: 1–19

Andreasen PA, Kjøller L, Christensen L and Duffy MJ (1997) The urokinase-type plasminogen activator system in cancer metastasis: a review. *Int J Cancer* **72**: 1–22

Behrendt N, Rønne E and Danø K (1995) The structure and function of the urokinase receptor, a membrane protein governing plasminogen activation on the cell surface. *Biol Chem Hoppe-Seyler* **376**: 269–279

Behrendt N, Rønne E and Danø K (1996) Domain interplay in the urokinase receptor; requirement for the third domain in high affinity ligand binding and demonstration of ligand contact sites in distinct receptor domains. *J Biol Chem* **271**: 22885–22894

Bouchet C, Hacène K, Martib PM, Becette V, Tubiana-Hulin M, Lasry S, Oglobine J and Spyratos F (1999) Dissemination risk index based on plasminogen activator system components in primary breast cancer. *J Clin Oncol* **17**: 3048–3057

Bradford MM (1976) A rapid and sensitive method for the quantitation of microgram quantities of protein utilizing the principle of protein-dye binding. *Anal Biochem* **72**: 248–254

Chapman HA (1997) Plasminogen activators, integrins, and the coordinated regulation of cell adhesion and migration. *Current Opinion Cell Biol*. **9**: 714–724

Danø K, Behrendt N, Brünner N, Ellis V, Ploug M and Pyke C (1984) The urokinase receptor: protein structure and role in plasminogen activation and cancer invasion. *Fibrinolysis* **8** (Suppl. 1): 189–203

Danø K, Andreasen PA, Grøndahl-Hansen J, Kristensen P, Nielsen LS and Skriver L (1985) Plasminogen activators, tissue degradation, and cancer. *Adv Cancer Res* **44**: 139–266

De Witte H, Pappot H, Brünner N, Grøndahl-Hansen J, Høyer-Hansen G, Behrendt N, Guldhammer-Skov B, Sweep F, Benraad Th and Danø K (1997) ELISA for complexes between urokinase-type plasminogen activator and its receptor in lung cancer tissue extracts. *Int J Cancer* **72**: 416–423

De Witte JH, Sweep CGJ, Klijn JGM, Grebenschikov NI, Peters HA, Look MP, Van Tienoven TH, Heuvel JJTM, Van Putten WLJ, Benraad ThJ and Foekens JA (1999a) Prognostic impact of urokinase-type plasminogen activator (uPA) and its inhibitor (PAI-1) in cytosols and pellet extracts derived from 892 breast cancer patients. *Br J Cancer* **79**: (7/8): 1190–1198

De Witte JH, Sweep CGJ, Klijn JGM, Grebenschikov N, Peters HA, Look MP, Van Tienoven ThH, Heuvel JJTM, Bolt-De Vries J, Benraad ThJ and Foekens JA (1999b) Prognostic value of tissue-type plasminogen activator (tPA) and its complex with the type-1 inhibitor (PAI-1) in breast cancer. *Br J Cancer* **80** (1/2): 286–294

Duffy MJ (1992) The role of proteolytic enzymes in cancer invasion and metastasis. *Clin Exp Metastasis* **10**: 145–155

Duffy MJ (1996) Proteases as prognostic markers in cancer. *Clin Cancer Res* **2**: 613–618

Duggan C, Maguire T, McDermott E, O'Higgins N, Fennelly JJ and Duffy MJ (1995) Urokinase plasminogen activator and urokinase plasminogen activator receptor in breast cancer. *Int J Cancer* **61**: 597–600

Ellis V, Behrendt N and Danø K (1991) Plasminogen activation by receptor-bound urokinase; a kinetic study with both cell-associated and isolated receptor. *J Biol Chem* **266**: 12752–12758

E.O.R.T.C. Breast Cancer Co-operative Group (1980) Revision of the standards for the assessment of hormone receptors in human breast cancer; report of the second E.O.R.T.C. workshop, held on 16–17 March, 1979, in The Netherlands Cancer Institute. *Eur J Cancer* **16**: 1513–1515

Foekens JA, Portengen H, Van Putten WLJ, Peters HA, Krijnen HLJM, Alexieva-Figusch J and Klijn JGM (1989) Prognostic value of estrogen and progesterone receptors measured by enzyme immunoassays in human breast tumor cytosols. *Cancer Res* **49**: 5823–5828

Foekens JA, Peters HA, Look MP, Portengen H, Schmitt M, Kramer MD, Brünner N, Jänicke F, Meijer-van Gelder M, Henzen-Logmans SC, van Putten WLJ and Klijn JGM (2000) The urokinase system of plasminogen activation and prognosis in 2780 breast cancer patients. *Cancer Res* **60**: 636–643

Grøndahl-Hansen J, Peters HA, Van Putten WLJ, Look MP, Pappot H, Rønne E, Danø K, Klijn JGM, Brünner N and Foekens JA (1995) Prognostic significance of the receptor for urokinase plasminogen activator in breast cancer. *Clin Cancer Res* **1**: 1079–1087

Kanse SM, Kost C, Wilhelm OG, Andreasen PA and Preissner KT (1996) The urokinase receptor is a major vitronectin-binding protein on endothelial cells. *Exp Cell Res* **224**: 344–353
Kaplan EL and Meier P (1958) Nonparametric estimation from incomplete observation. *J Am Stat Assoc* **53**: 457–481
Kristensen P, Eriksen J, Blasi F and Danø K (1991) Two alternatively spliced mouse urokinase receptor messenger RNAs with different histological localization in the gastrointestinal tract. *J Cell Biol* **115**: 1763–1771
Liotta LA, Thorgeirsson UP and Garbisa S (1982) Role of collagenases in tumor cell invasion. *Cancer Metastasis Rev* **1**: 277–288
Look MP and Foekens JA (1999) Clinical relevance of the urokinase plasminogen activator system in breast cancer. APMIS **107 (1)**: 150–159
Mignatti P and Rifkin DB (1993) Biology and biochemistry of proteinases in tumour invasion. *Physiol Rev* **73**: 161–195
Pappot H, Høyer-Hansen G, Rønne E, Hansen HH, Brünner N, Danø K and Grøndahl-Hansen J (1997) Elevated plasma levels of urokinase plasminogen activator receptor in non-small cell lung cancer patients. *Eur J Cancer* **33**: 867–872
Ploug M, Rønne E, Behrendt N, Jensen AL, Blasi F and Danø K (1991) Cellular receptor for urokinase plasminogen activator; carboxyl-terminal processing and membrane anchoring by glycosyl-phosphatidylinositol. *J Biol Chem* **266**: 1926–1933
Ploug M, Eriksen J, Plesner T, Hansen NE and Danø K (1992) A soluble form of the glycolipid-anchored receptor for urokinase-type plasminogen activator is secreted from peripheral blood leukocytes from patients with paroxysmal nocturnal hemoglobinuria. *Eur J Biochem* **208**: 397–404
Pyke C, Eriksen J, Solberg H, Nielsen BS, Kristensen P, Lund LR and Danø K (1993) An alternatively spliced variant of mRNA for the human receptor for urokinase plasminogen activator. *FEBS Lett* **326**: 69–74
Rønne E, Behrendt N, Ploug M, Nielsen HJ, Wöllisch E, Weidle U, Danø K and Høyer-Hansen G (1994) Quantitation of the receptor for urokinase plasminogen activator by enzyme-linked immunosorbent assay. *J Immunol Methods* **167**: 91–101
Rønne E, Høyer-Hansen G, Brünner N, Pedersen H, Rank F, Osborne CK, Clark GM, Danø K and Grøndahl-Hansen J (1995) Urokinase receptor in breast cancer tissue extracts; enzyme-linked immunosorbent assay with a combination of mono- and polyclonal antibodies. *Breast Cancer Res Treat* **33**: 199–207
Schmitt M, Harbeck N, Thomssen C, Wilhelm O, Magdolen V, Reuning U, Ulm K, Höfler H, Jänicke F and Graeff H (1997) Clinical impact of the plasminogen activation system in tumor invasion and metastasis: prognostic relevance and target for therapy. *Thromb Haemost* **78**: 285–296
Sier CF, Stephens R, Bizik J, Mariani A, Bassan M, Pedersen N, Frigerio L, Ferrari A, Dano K, Brunner N and Blasi F (1998) The level of urokinase-type plasminogen activator receptor is increased in serum of ovarian cancer patients. *Cancer Res* **58**: 1843–1849
Stephens RW, Pedersen AN, Nielsen HJ, Hamers MJAG, Høyer-Hansen G, Rønne E, Dybkjær E, Danø K and Brünner N (1997) ELISA determination of soluble urokinase receptor in blood from healthy donors and cancer patients. *Clin Chem* **43**: 1868–1876
Stephens RW, Nielsen HJ, Christensen IJ, Thorlacius-Ussing O, Sorensen S, Danø K and Brünner N (1999) Plasma urokinase receptor levels in patients with colorectal cancer: relationship to prognosis *J Natl Cancer Inst* **91(10)**: 869–874
Waltz DA and Chapman HA (1994) Reversible cellular adhesion to vitronectin linked to urokinase receptor occupancy. *J Biol Chem* **269**: 14746–14750
Wei Y, Waltz DA, Rao N, Drummond RJ, Rosenberg S and Chapman HA (1994) Identification of the urokinase receptor as an adhesion receptor for vitronectin. *J Biol Chem* **269**: 32380–32388
Wei Y, Lukashev M, Simon DI, Bodary SC, Rosenberg S, Doyle MV and Chapman HA (1996) Regulation of integrin function by the urokinase receptor. *Science* **273**: 1551–1555

© 2001 Cancer Research Campaign
